# Association Between Peripapillary Bruch's Membrane Shape and Intracranial Pressure: Effect of Image Acquisition Pattern and Image Analysis Method, a Preliminary Study

**DOI:** 10.3389/fneur.2018.01137

**Published:** 2018-12-21

**Authors:** Kiran Malhotra, Megh D. Patel, Zainab Shirazi, Heather E. Moss

**Affiliations:** ^1^Department of Ophthalmology & Visual Sciences, University of Illinois at Chicago, Chicago, IL, United States; ^2^Department of Ophthalmology, Stanford University, Palo Alto, CA, United States; ^3^Department Neurology & Neurological Sciences, Stanford University, Stanford, CA, United States

**Keywords:** intracranial pressure, Bruch's membrane, OCT, idiopathic intracranial hypertension, geometric morphometric analysis

## Abstract

**Background/Aims:** High intracranial pressure (ICP) is associated with changes in peripapillary Bruch's membrane (pBM) shape on optical coherence tomography (OCT) images of the optic nerve head. It is not known if image acquisition pattern and analysis method impact this association.

**Materials and Methods:** Cross sectional OCT scans of the optic nerve head were obtained at six angles using a radial scan pattern in 21 subjects immediately prior to ICP measurement via lumbar puncture. On each image, Bruch's membrane was manually segmented and defined by either 14 or 16 semi-landmarks and either rater identified, or distance identified boundaries. For each of these four image analysis strategies, geometric morphometric analysis identified the first principal component of Bruch's membrane shape for all images and for the set of images taken at each angle. Repeated measures ANOVA of the first principal component magnitude (PC1) for all images assessed for shape difference between image angles. Linear generalized estimating equation models assessed association between angle specific first principal component magnitudes (PC1) and ICP for each angle. Receiver operating characteristic analysis assessed angle specific PC1s' ability to differentiate elevated from normal ICP.

**Results:** The first principal component represented deflection into the vitreous for all scan angles, but quantitatively differed across scan angles (*p* < 0.005, repeated measures ANOVA). Angle specific first principal components were positively correlated with ICP (*p* < 0.005 for all angles, generalized estimating equation models). All angle specific first principal components showed excellent ability to classify ICP (area under curve ≥ 0.8 for all). These results were independent from image analysis strategy.

**Discussion:** Though qualitative changes in Bruch's membrane shape are similar regardless of cross-sectional angle of the 2-D OCT scan, they differ quantitatively between OCT scan angles, meaning that pBM is not axially symmetric and therefore PC1 extracted from different 2-D scan angles can't be compared between individuals. However, we do not identify an optimal scan angle for classification of ICP since there is a similarly strong linear relationship between the first principal component of shape and ICP and angle specific first principal components of Bruch's membrane shape showed similarly excellent ability to differentiate elevated from normal ICP. The results support development of Bruch's membrane shape extracted from 2-D cross sectional optic nerve head OCT scans as a biomarker of ICP and emphasize the importance of consistency of scan angle. This is relevant for developing diagnostic protocols that use OCT to detect high ICP states.

## Introduction

Idiopathic intracranial hypertension (IIH) affects 1:100,000 individuals annually with a 20-fold higher incidence in young, obese females (1, 2). In this condition, papilledema, or swelling of the optic nerve head, occurs in response to the exposure of optic nerve axons and their vascular supply to elevated intracranial pressure (ICP) in the optic nerve sheath (3, 4). Visual impairment is a major morbidity of IIH. Physicians currently use downstream outcomes, such as peripheral vision loss and optic disk appearance to monitor patients, though changes in these outcomes can take days to manifest following ICP change (5). A promising marker that may reflect ICP change in a more timely fashion is deformation of the ocular globe around the optic nerve, which deflects into the vitreous cavity in states of high ICP and can be visualized by superior deflection of peripapillary Bruch's membrane (pBM) opening on optical coherence tomography (OCT) scans.

**Figure 1:**
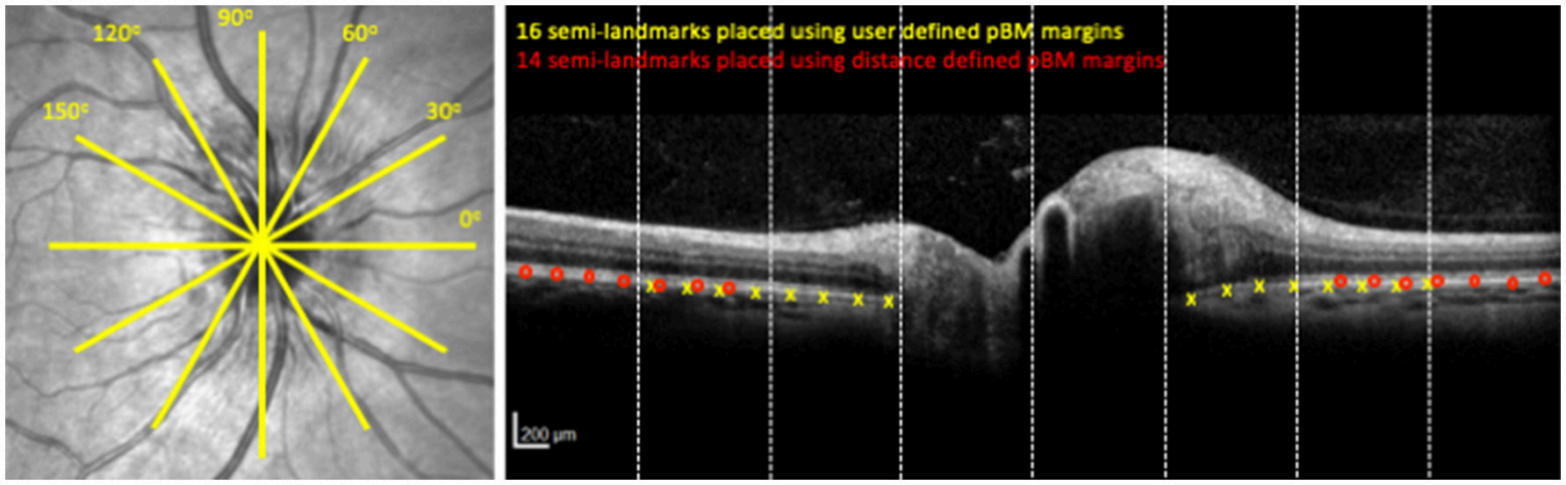
OCT image acquisition pattern and image analysis. Left panel shows the location of cross sectional OCT scans through the optic nerve head. Each yellow line is a cross section. Right panel shows the OCT cross sectional image at 0° for the optic nerve head shown on the left. Yellow x's indicate semi-landmark placement using the User-16 strategy. Red circles indicate semi-landmark placement using Distance-14 strategy. Dashed vertical lines show boundaries of segments for purposes of image reliability assessment.

The majority of prior studies that support the development of pBM shape as a marker of ICP have been based on 2 dimensional analysis of a single cross-sectional OCT B-scan of the optic nerve head oriented along the horizontal nasal/temporal axis (Figure 1) (6–9). Study of other scan angles is relevant because the optic nerve head is not a radially symmetric structure (10). The first aim of this study is to test the hypothesis that the pBM shape-ICP relationship is impacted by scan angle and to identify the optimal scan angle for differentiating high from normal ICP states.

A challenge identified in prior studies of pBM shape ICP relationships has been identifying the margins of the pBM opening, which can be difficult when there is shadowing of the area due to optic nerve head swelling (9, 11, 12). The second aim of this study is to compare image analysis strategies for defining pBM with regards to whether they affect the relationship between pBM shape and ICP.

## Materials and Methods

### Subjects

As part of a study of ophthalmic imaging associations with intracranial pressure, 21 adults (age 23–86 years) scheduled to undergo lumbar puncture for a clinical indication were recruited from the clinical practices of the University of Illinois at Chicago. Demographic information and lumbar puncture parameters (reason for performing, position, opening pressure) were collected for each subject. Based on the opening pressure during lumbar puncture, subjects' ICP was categorized as normal ( ≤ 20 cm H_2_O), borderline (20 < ICP < 25 cm H_2_O) or elevated ICP (≥25 cm H_2_O). The study was approved by the Institutional Review Board at the University of Illinois at Chicago and was conducted in accordance to the Declaration of Helsinki in its current form. The nature and possible consequences of the study were explained to each subject, and written consent was obtained.

### Image Aquisition

Six OCT B-scans of the optic nerve head were obtained using a radial scan pattern centered on the optic nerve head (Spectralis, Heidelberg Engineering, Germany) (Figure 1) in both eyes within 1 h prior to lumbar puncture. The scans were 20° (1,024 pixels) in length with a resolution of 5.5–6.4 μm/pixel along the x-axis and axial resolution (*y*-axis) of 3.9 μm/pixel. Scan angles were in 30° increments labeled counter clockwise from nasal in right eyes and clockwise from nasal in left eyes. In these coordinates, 0° is the nasal-temporal scan, and 90° is a vertical scan.

### Image Segmentation

Left eye images were inverted on a vertical axis to align the temporal-nasal orientation of left and right eyes. Two raters independently used customized software (MatLab, Mathworks Inc., Natick, MA, USA) to manually segment pBM on either side of the optic nerve as a curved line with a discontinuity underneath the optic nerve head (12–14). The coordinates of the pBM curves were compared between raters to identify images with inter-rater differences ≥3 pixels in the axial (y) axis in each of eight equal sections in the *x* dimension (Figure 1). These sections were independently re-segmented following consensus review of the images by the raters to identify sources of disagreement. Sections and scan angle were compared to identify regions with most disagreement and the extent of disagreement. For each image the pBM curve was calculated as the mean axial (y) location for the two rateres for each given radial (x) location. For subsequent analysis, coordinates of the pBM curve were then scaled from pixels to microns using the scaling factors for each image contained in the image information provided by the device.

### Geometric Morphometric Analysis of Peripapillary Bruch's Membrane Shape

Geometric morphometric analysis, which characterizes shape independent from scale, translation, and rotation, has previously been applied to analyze peripapillary Bruch's Membrane (pBM) shape in varying states of ICP (6–9). The reader is referred elsewhere for full details of the general technique (15). Briefly, this analysis is performed using a set of geometric objects. Each object is defined using semi-landmarks placed in a consistent manner. The cartesian coordinates of the semi-landmarks for each object are the input for the analysis. A consensus shape is defined as the average position of each semi-landmark. Principal components, which are linearly independent combinations of each semi-landmark coordinate, are calculated to explain the variation in shape of the objects relative to the consensus shape. A principal component describes relative variation of the semi-landmarks along a vector for each object. The vector directions for a principal component are constant across objects. The magnitude of each principal component is specific to each object and indicates how far along that principal component's vector that object's shape is relative to the consensus shape.

In prior studies of pBM shape in high ICP the first principal component vector has represented deflection of the pBM margins into and away from the vitreous and the magnitude of the first principal component has represented the relative deflection into and away from the vitreous for a given image (6–9). Prior studies have define pBM using 16 semi-landmarks spaced at 180 μm increments away from rater identified pBM margins (Figure 1). In this study we used this technique as well as three other strategies for defining pBM. The other strategies varied in two ways. For two strategies the placement of the pBM margin markers was based on the distance of the optic nerve center rather than where the raters judged the margin to lie. This was done to exclude analysis of the regions of pBM for which there was the highest amount of rater disagreement occurred. In this strategy, pBM margins were placed 1,612 μm from the optic nerve center on each side. Second, for two strategies 14 instead of 16 semi-landmarks were used as some images could not accommodate eight semi-landmarks on each side of the pBM opening using distance specified margins. We selected 14 semi-landmarks in order to include the most images. We refer to the image analysis strategies according to their BM margin definition and the number of semi-landmarks: user-16, user-14, distance-16, distance-14. For purposes of comparison, analyses were done on both all eligible images as well as the subset that had successful semi-landmark placement using all four strategies.

Geometric morphometric analysis was completed using morphometrics freeware (SUNY, Stony Brook, NY, USA) (16). For each image analysis strategy (user-16, user-14, distance-16, distance-14), the principal components (PC) of shape for a set of images were calculated for the entire set of images (all eyes, all angles) and for each individual angle (all eyes, one angle). The first principal component (PC1) accounts for a majority of the shape variation across scans in a given image set and the magnitude of PC1 for a set of pBM images was previously shown to be associated with ICP in 0° (nasal-temporal) scans (6). Therefore, the PC1 magnitude was used as the primary outcome variable in this study. PC1_all_ refers to the first principal component magnitude for the set of all images and PC1_angle_ magnitude refers to the first principal component for the images at one angle.

### Statistical Analysis

Statistical analyses were performed using SPSS v24.0 (IBM Inc., Armonk, NY, USA). *P* < 0.05 established statistical significance. PC1_all_ (i.e., the PC1 magnitude for the image set comprised of all images) was compared between scan angles and eyes with ICP as a covariate using repeated measures ANOVA to test for associations between PC1_all_ and scan angle and for PC1_all_ and ICP.

PC1_0_, PC1_30_, PC1_60_, PC1_90_, PC1_120_, PC1_150_ (i.e., the PC1 magnitude for image sets comprised of images at a given scan angle) were modeled using linear generalized estimating equations (GEE), accounting for within-subject correlations, with ICP as the independent variable to evaluate for associations between ICP and pBM shape at each scan angle. Area under curve (AUC) calculated using receiver operating characteristic (ROC) analyses for PC1_0_, PC1_30_, PC1_60_, PC1_90_, PC1_120_, PC1_150_ were used to assess the ability of each angle specific PC1 to discriminate elevated (≥25 cm H_2_O) from normal or borderline (< 25 cm H_2_O) ICP (i.e., excluding borderline ICP) and to discriminate normal (≤ 20 cm H_2_O) from borderline or high ICP (>20 cm H_2_O). Only one eye per subject was included in this ROC analysis so that the observations were independent.

## Results

### Subjects

Thirty-five subjects were recruited, and 21 completed the study (Table 1). The remaining 14 were excluded due to failed LP. Data from one eye of one subject were excluded because 16 semi-landmarks could not be defined on any scan. Therefore, 246 B-scans from 41 eyes were analyzed.

**Table 1:** Subject features.

Age	42.4 ± 16.9 years
Lumbar Puncture Opening Pressure	26.5 ± 12.4 cm H_2_O
**OPENING PRESSURE CATEGORY**	
Normal ICP (≤ 20 cm H_2_O)	5 (24%)
Borderline ICP (20 < ICP < 25 cm H_2_O)	5 (24%)
Elevated ICP (≥25 cm H_2_O)	11 (52%)
**REASON FOR LP**	
Dementia	4 (19%)
Headache	9 (43%)
Inflammation	7 (33%)

*Continuous variable table entries are average ± standard deviation, categorical variable table entries are n (%)*.

### Image Segmentation Reliability

For purposes of reliability analysis, each image was divided into eight equal horizontal segments (Figure 1). 228 of 1,968 (12%) image segments had one or more locations on the x-axis with a 3 pixel (11.7 μm) or greater difference in axial (*y* axis) segmentation between raters. These were disproportionately distributed in the central (peripapillary) image sections, where 33% of images had at least one location differing by 3 or more pixels between raters. In comparison only 4.3% of outer sections had at least one location differing by the same amount. Among locations with intra-rater disagreement of 3 or more pixels, the magnitude of the difference was right skewed [range 3–31 pixels, mean 4.5 pixels (18 μm)]. Disagreement proportions did not differ across scan angles (*p* > 0.05, ANOVA). Following consensus review and repeat segmentation, proportion of segments with 3 or more pixels of disagreement was reduced to 9% overall and average of the pixel difference of points exceeding the intra-rater difference threshold was reduced to an average 3.6 pixels (14 μm) with a maximum difference of 13 pixels only seen in the peripapillary sections.

### Geometric Morphometric Shape Analysis

**Figure 2:**
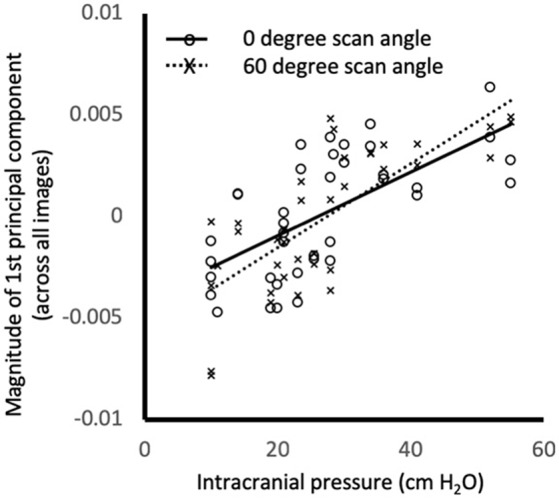
Relationship between intracranial pressure (measured as opening pressure during lumbar puncture) and magnitude of the first principal component of shape for the all images. Markers show the values for individual images at 0° (o) and 60° (x) using distance-14 semi-landmarks. Lines are linear regression fits to 0° (solid) and 60° (dashed). PC1_all_ magnitude was similar between left and right eyes, but differed across scan angles accounting for ICP (*p* < 0.005, rmANOVA). Similar relationships were observed for different scan angles and semi-landmark placement strategies (not shown).

Semi-landmarks were defined using the user-16 and user-14 strategies on all 246 images. For distance-16 and distance-14 strategies, semi-landmarks could be placed on 196 images and 238 images, respectively. For all image analysis strategies PC1_all_ magnitude was similar between left and right eyes, but differed across scan angles accounting for ICP (*p* < 0.005, rmANOVA for user-16, distance-16, user-14, and distance-14 image analysis strategies, Figure 2).

**Figure 3:**
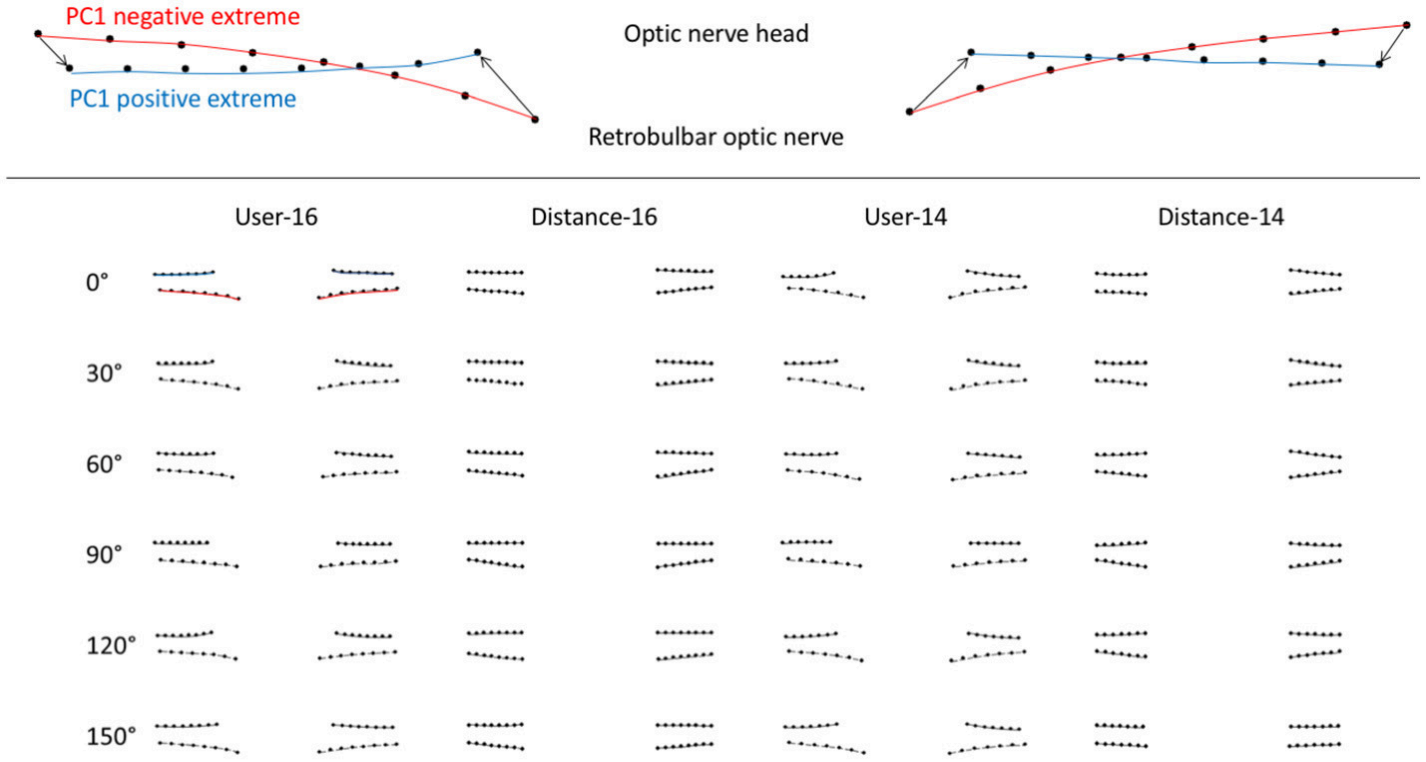
Scan angle specific first principal components of peripapillary Bruch's membrane shape according OCT acquisition pattern (scan angle, rows) and image analysis strategy (columns) calculated using geometric morphometric analysis. Upper image is an example of a first principal component of pBM shape. The red curves represent the negative extreme (−1) of the PC and the blue curves represent the positive extreme (+1). The markers are the semi-landmarks for that extreme. The arrows between semi-landmarks (only 4 shown for simplicity) are the trajectories of semi-landmarks for the PC illustrated. Images in the table shows pBM shapes for the extreme negative (lower) and positive (upper) magnitudes of PC1 for images taken at a particular scan angle (row) and analyzed using a particular strategy (column).

PC1_angle_ represented deflection of pBM into/out of the eye for all image analysis strategies (Figure 3). PC1_angle_ calculated using user-16 and user-14 also captured widening of the BM opening associated with deflection into the eye, while those calculated using distance-16 and distance-14 did not (Figure 3). ICP was linearly associated with PC1_angle_ magnitudes for each scan angle regardless of method of image analysis (*p* < 0.0005–0.03, GEE for all angles and all image analysis strategies). For the subset of images for which semi-landmarks could be defined using all strategies the relationship between ICP and PC1_angle_ were similar (*p* < 0.0005–0.026, GEE for all angles, and all image analysis strategies).

**Figure 4:**
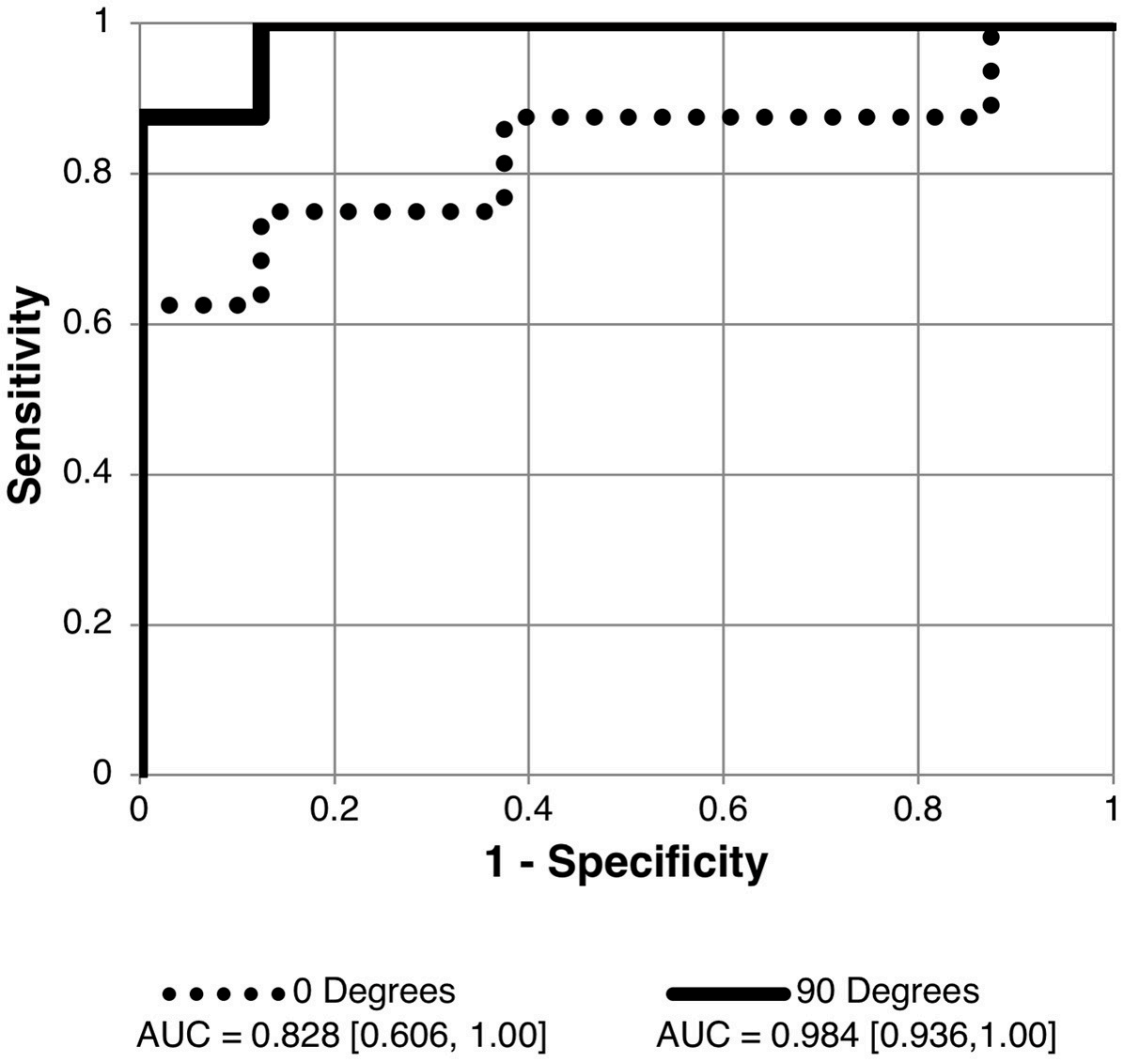
Receiver operating characteristic (ROC) curve for detection of elevated intracranial pressure (ICP ≥ 25 cm H_2_O measured as lumbar puncture opening pressure) using angle specific first principal component for pBM shape. Curves shown for 90 and 0° scan angles using user-16 method of semi-landmark placement are shown. AUC values and confidence intervals shown below chart. See Table 2 for AUC results for other scan angles and image analysis strategies.

**Figure 5:**
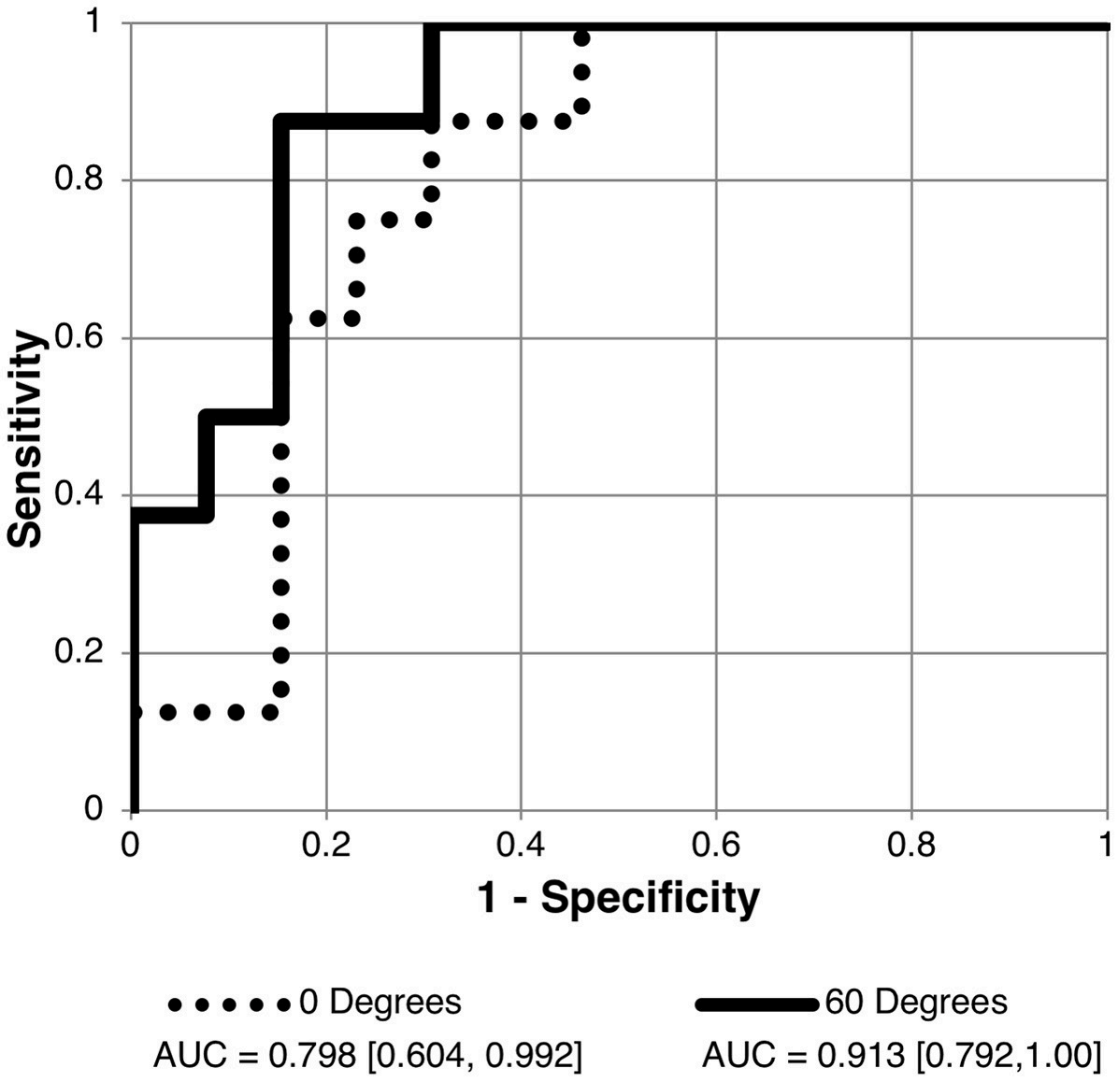
Receiver operating characteristic (ROC) curve for detection of normal intracranial pressure (ICP ≤ 20 cm H2O measured as lumbar puncture opening pressure) using the scalar of the angle specific first principal component for pBM shape. Curves shown for 90 and 60° scan angles using user-16 method of semi-landmark placement. AUC values and confidence intervals shown below chart. See Table 3 for AUC results for other scan angles.

Receiver operating characteristic analyses were used to assess PC1_angle_ ability to differentiate normal or borderline ICP from elevated ICP and to differentiate normal from elevated or borderline ICP. ROC analyses demonstrated excellent ability for PC1_angle_ magnitudes to differentiate elevated ICP from normal or borderline ICP (ICP ≥ 25 vs. < 25 cm H_2_O, Table 2, Figure 4) and to differentiate normal from elevated or borderline ICP (ICP ≤ 20 vs. >20 cm H_2_O, Table 3, Figure 5). 95% confidence intervals for all PC1_angle_ AUC overlapped suggesting similar differentiating ability.

**Table 2:** Receiver operating characteristic analysis for detection of elevated ICP (≥25 cm H_2_O) using the magnitude of the angle specific-first principal component of peripapillary Bruch's membrane shape.

	**Semi-landmark placement strategy**			
**Scan Angle**	**User-16**	**User-14**	**distance-14**	**distance-16**
0	0.828 [0.606, 1.00]	0.813 [0.581, 1.00]	0.875 [0.694, 1.00]	0.918 [0.754, 1.00]
30	0.906 [0.727, 1.00]	0.938 [0.808, 1.00]	0.875 [0.694, 1.00]	0.918 [0.754, 1.00]
60	0.969 [0.892, 1.00]	0.938 [0.822, 1.00]	0.813 [0.571, 1.00]	0.898 [0.702, 1.00]
90	0.984 [0.936, 1.00]	0.984 [0.936, 1.00]	0.922 [0.784, 1.00]	0.959 [0.861, 1.00]
120	0.984 [0.936, 1.00]	0.984 [0.936, 1.00]	0.906 [0.727, 1.00]	0.881 [0.655, 1.00]
150	0.906 [0.727, 1.00]	0.906 [0.727, 1.00]	0.922 [0.784, 1.00]	0.857 [0.644, 1.00]

*Rows are OCT scan angle through the optic nerve head. Cell entries are area under curve point estimate (95% confidence interval) for classification of high (≥25 cm H2O) vs. normal or borderline (< 25 cm H_2_O) ICP*.

**Table 3:** Receiver operating characteristic analysis results for detection of normal ICP (≤ 20 cm H_2_O) using the magnitude of the angle specific-first principal component of peripapillary Bruch's membrane shape.

	**Semi-landmark placement strategy**			
**Scan Angle**	**user-16**	**user-14**	**distance-14**	**distance-16**
0	0.798 [0.604, 0.992]	0.788 [0.591, 0.986]	0.846 [0.680, 1.00]	0.883 [0.719, 1.00]
30	0.827 [0.639, 1.00]	0.846 [0.670, 1.00]	0.846 [0.680, 1.00]	0.883 [0.719, 1.00]
60	0.913 [0.792, 1.00]	0.885 [0.741, 1.00]	0.798 [0.602, 0.994]	0.870 [0.692, 1.00]
90	0.894 [0.758, 1.00]	0.885 [0.741, 1.00]	0.875 [0.725, 1.00]	0.935 [0.825, 1.00]
120	0.875 [0.722, 1.00]	0.865 [0.707, 1.00]	0.894 [0.748, 1.00]	0.871 [0.692, 1.00]
150	0.827 [0.647, 1.00]	0.817 [0.630, 1.00]	0.885 [0.743, 1.00]	0.857 [0.681, 1.00]

*Cell entries area under curve point estimate (95% confidence interval) for classification of normal (≤ 20 cm H2O) vs. high or borderline (> 20 cm H_2_O) ICP*.

## Discussion

In follow-up to prior studies showing association between peripapillary Bruch's membrane shape measured using two-dimensional OCT B-scan shape and intracranial pressure (6–8), we sought to determine if OCT image acquisition pattern and image analysis strategy impact the relationship between pBM shape and intracranial pressure. Specifically, we studied the effect of angle of OCT scan through the optic nerve head and strategy of semi-landmark placement for geometric morphometric analysis.

We found that qualitative pBM shape is similar across scan angles, reflecting deflection of pBM margins into the vitreous with increasing levels of ICP. However, quantitatively pBM shape differs, supporting the notion that pBM is not an axially symmetric. The implication of this observation is that it is not valid to compare quantitative shape parameters extracted from different scan angles across subjects and that care must be taken in any future study to have scan angle consistency.

Though pBM shape varies between scan angles, pBM shape was linearly associated with ICP for each angle studied. Furthermore, the magnitudes of the first principal component of shape for each scan angle performed similarly for differentiating between ICP states. Thus, we did not identify an optimal scan angle with which to characterize ICP on the basis of pBM shape. These findings have application to developing pBM shape for use in diagnosing and monitoring ICP in clinical practice in that any scan angle can be used so long as it is consistent between subjects. It would be reasonable to select scan angle based on imaging acquisition consistency and quality. Further work is needed to determine if three-dimensional analysis offers any advantage over two-dimensional analysis, which is computationally more efficient.

Accurate and reliable analysis of OCT images is critical as it is the input for pBM shape analysis. While manual segmentation of pBM can be laborious for larger datasets, Iverson et al suggest that pBM automatic segmentation programs may inaccurately identify the pBM end point when it is close to the cup border, when the border tissue of Bruch's membrane extends past the RPE or when the signal intensity is diminished from shadows generated by overlying vasculature in non-swollen optic nerves (17). In the case of swollen optic nerves, this challenge, also reported by other investigators, is likely due to artifact, and decreased contrast in this area of the image due to decreased light penetration through optic nerve head tissue (12, 18). We confirmed that the portions of pBM adjacent to the BM under the optic nerve have the most disagreement between raters performing manual segmentation. While consensus review followed by resegmentation was effective in improving reliability, it did not address the concern of validity regarding identification of the actual pBM margin. A strategy to circumvent this is to exclude this region from analysis by placing the central semi-landmarks by distance from the optic nerve center. This modified strategy was straightforward to implement and impacted neither the nature of relationships between pBM shape and ICP nor the ability or pBM shape to differentiate between ICP states. We conclude that placement of the margin landmarks by distance improves landmark placement reliability without sacrificing classification ability of shape metrics.

Limitations to this study include that subjects with normal ICP had other neurological conditions that prompted lumbar puncture. The “gold standard” ICP measure was a point measure via lumbar puncture, which may not accurately reflect current or steady state CSF pressure in the optic nerve sheath. While this selection bias may limit generalizability of results with regards to predicting ICP, it is unlikely to impact the conclusions regarding scan angle or pBM shape definition methodology. Sample size is a limitation.

In conclusion, manual segmentation reliability of the pBM layer improves between raters after consensus review and does not differ based on scan angle. Excluding regions of pBM prone to disagreement did not impact pBM shape vs. ICP relationships. We found pBM shape to be related to ICP and to have excellent ability to classify ICP state regardless of scan angle or semi-landmark placement strategy. A larger prospective study is warranted to train and validate an ICP classifier based on pBM shape and to determine the role of covariates, such as axial length, age and gender. Automation of image segmentation and landmark placement would be ideal for such an investigation.

## Author Contributions

KM and HM conceived and designed the analysis. ZS collected the data. KM, MP, and HM performed the analysis. KM, MP, ZS, and HM wrote the paper.

### Conflict of Interest Statement

The authors declare that the research was conducted in the absence of any commercial or financial relationships that could be construed as a potential conflict of interest. The reviewer JK declared a shared affiliation, though no other collaboration with several of the authors KM, ZS to the handling Editor.
